# Annual acknowledgement of manuscript reviewers

**DOI:** 10.1186/1748-5908-8-100

**Published:** 2013-09-11

**Authors:** Anne Sales, Michel Wensing

**Affiliations:** 1 Co-Editors-in-Chief, Implementation Science

## Abstract

**Contributing reviewers:**

The editors of *Implementation Science* would like to thank all our reviewers who have contributed to the journal in Volume 7 (2012).

## 

Charles Abraham

United Kingdom

Sara Ackerman

United States of America

Susan Adams

United States of America

Mohamed Ahmedna

Qatar

Bianca Albers

Denmark

Michael Allen

Canada

Elaine Amella

United States of America

Peter Anderson

Spain

Lou Atkins

United Kingdom

Ross Bailie

Australia

Richard Baker

United Kingdom

Luciana Ballini

Italy

Daynia Elizabeth Ballot

South Africa

Claire Bamford

United Kingdom

Jonathan Benn

United Kingdom

Fiona Beyer

United Kingdom

Barbara Bokhour

United States of America

Gary Bond

United States of America

Andrew Booth

United Kingdom

Cameo Borntrager

United States of America

Alan Borthwick

United Kingdom

Marije Bosch

Australia

Candice Bowman

United States of America

Gudrun Boysen

Denmark

Jeffrey Braithwaite

Australia

Joze Braspenning

Netherlands

Jamie Brehaut

Canada

Sue Brennan

Australia

Melissa Brouwers

Canada

C Hendricks Brown

United States of America

Richard Byng

United Kingdom

Guy Cafri

United Kingdom

James Cane

United Kingdom

Tim Carey

Australia

Rosemary Caron

United States of America

Enrique Castro-Sanchez

United Kingdom

Cristina Catallo

Canada

Thane Chambers

Canada

Esmita Charani

United Kingdom

Francine Cheater

United Kingdom

Kath Checkland

United Kingdom

Helen Chin

United Kingdom

Siriporrn Chirawatkul

Thailand

Erika Frischknecht Christensen

Denmark

Theopisti Chrysanthaki

United Kingdom

Alexander Clark

Canada

Sean Clarke

United States of America

Stephanie Cohen

United States of America

Heather Colquhoun

Canada

Simon Coulton

United Kingdom

Luis Gabriel Cuervo

United States of America

Nicky Cullum

United Kingdom

Gavin Daker-White

United Kingdom

Laura Damschroder

United States of America

Frank Davidoff

United States of America

Mark Davis

Australia

Carl de Wet

United Kingdom

James Dearing

United States of America

Vincent Deary

United Kingdom

Sophie Desroches

Canada

Brian Dixon

United States of America

Mary Dixon-Woods

United Kingdom

Maureen Dobbins

Canada

Debra Dobbs

United States of America

Andrew Don-Wauchope

Canada

Shannon Dorsey

United States of America

Colleen Doyle

Australia

Pierre Durieux

France

Judith Dyson

United Kingdom

Mark Ebell

United States of America

Andrew Elders

United Kingdom

Ann Catrine Eldh

Sweden

Mike English

Kenya

Anna Essén

Sweden

Tracy Finch

United Kingdom

Gerd Flodgren

United Kingdom

Jay Ford

United States of America

Jill Francis

United Kingdom

Atle Fretheim

Norway

Jeffrey Fuller

Australia

Anna Gagliardi

Canada

Nicola Gale

United Kingdom

Afschin Gandjour

Germany

Benjamin Gardner

United Kingdom

Ann Garland

United States of America

Max Geraedts

Germany

Russell Glasgow

United States of America

Paul Glasziou

Australia

Lisa Gold

Australia

Ralph Gonzales

United States of America

Ian Graham

Canada

Gonzalo Grandes

Spain

Sarah Griffin

United States of America

Iñaki Gutierrez

Spain

Mary Haines

Australia

Carmen Hall

United States of America

Kara Hall

United States of America

Jonathan Hammond

United Kingdom

Margaret Handley

United States of America

Wendy Hardeman

United Kingdom

Katherine Harding

Australia

Claire Harris

Australia

Margaret B. Harrison

Canada

Gill Harvey

United Kingdom

Penelope Hawe

Canada

Larry Hearld

United States of America

Robert Hecht

United States of America

Anthony Hemmelgarn

United States of America

Susanne Hempel

United States of America

Jane Hendy

United Kingdom

Anne Holland

Australia

Margaret Holmes-Rovner

United States of America

Daniel Holt

United States of America

Michelle Honey

New Zealand

Maria Horne

United Kingdom

Alison Hutchinson

Australia

Sylvia Hysong

United States of America

Irene Ilott

United Kingdom

Theodore Iwashyna

United States of America

Catherine Jackson

United Kingdom

Jesse Jacobs

United States of America

Katharina Janus

United States of America

Bonnie Jennings

United States of America

Patrick Jeurissen

Netherlands

Paul Johnson

Australia

Marie Johnston

United Kingdom

Lydia Kapiriri

Canada

Elizabeth Kendall

Australia

Amy Kilbourne

United States of America

Gad Kilonzo

Tanzania

Roman Kislov

United Kingdom

Alison Kitson

United Kingdom

Niina Kolehmainen

United Kingdom

Tiina Kortteisto

Finland

Anita Kothari

Canada

Saravana Kumar

Australia

Regina Kunz

Switzerland

Joseph Kwan

United Kingdom

Yiannis Kyratsis

United Kingdom

Anna Lau

United States of America

Miranda Laurant

Netherlands

Rebecca Lawton

United Kingdom

Jennifer Leeman

United States of America

France Legare

Canada

Laura Leviton

United States of America

Cara Lewis

United States of America

Linda Li

Canada

Andrew Lockett

United Kingdom

Lisa Lubomski

United States of America

Pisake Lumbiganon

Thailand

Renee Lyons

Canada

Annette Majnemer

Canada

Richard Martin

United States of America

Graham Martin

United Kingdom

Carl May

United Kingdom

Danielle Mazza

Australia

John McAteer

United Kingdom

Julie McDonald

Australia

Rosemary McEachan

United Kingdom

Bradford McFadyen

Canada

Katherine McGilton

Canada

Lorna McKee

United Kingdom

Ann McKibbon

Canada

Fiona Miller

Canada

Stephen Mills

Thailand

David Moher

Canada

Gerard Molloy

United Kingdom

Gabriel Moore

Australia

Donna Moralejo

Canada

Jill Morrison

United Kingdom

Felix Naughton

United Kingdom

Skye Newton

Australia

Per Nilsen

Sweden

Wynne Norton

United States of America

Sandra Nutley

United Kingdom

Mathieu Ouimet

Canada

Deborah Parker

Australia

John Parsons

New Zealand

Salvador Peiró

Spain

David Pilgrim

United Kingdom

Byron Powell

United States of America

Peter Pronovost

United States of America

Joanne Protheroe

United Kingdom

Jeff Pyne

United States of America

Craig Ramsay

United Kingdom

Geetha Ranmuthugala

Australia

Tim Rapley

United Kingdom

Arash Rashidian

Iran

Melody Rhydderch

United Kingdom

JaMuir Robinson

United States of America

Hector Rodriguez

United States of America

Sarah Rosenbaum

Norway

Patricia Rosenberger

United States of America

Lisa Rubenstein

United States of America

Bruno Rushforth

United Kingdom

Jo Rycroft-Malone

United Kingdom

Anna Sallis

United Kingdom

Shannon Scott

Canada

Nick Sevdalis

United Kingdom

Rod Sheaff

United Kingdom

Paul Shekelle

United States of America

Sasha Shepperd

United Kingdom

Nahoko Shindo

Switzerland

Stephanie Short

Australia

Jane Silovsky

United States of America

Sara Singer

United States of America

Falko F Sniehotta

United Kingdom

Leif Solberg

United States of America

Nelson SooHoo

United States of America

Neil Spicer

United Kingdom

Katherine Stamatakis

United States of America

Nicholas Steel

United Kingdom

Cheryl Stetler

United States of America

Keith Stevenson

United Kingdom

Frances Stillman

United States of America

Tim Stokes

United Kingdom

Sharon Straus

Canada

Natalie Taylor

United Kingdom

George Teather

Canada

Alan Thomas

United Kingdom

Eric Thomas

United States of America

Edel Tierney

Ireland

Jonathan Tobin

United States of America

Chris Todd

United Kingdom

Lyndal Trevena

Australia

Tari Turner

Australia

Sara Twaddle

United Kingdom

Trudy van der Weijden

Netherlands

Charles Vincent

United Kingdom

Ivo Vlaev

United Kingdom

Tara Vlasimsky

United States of America

Dorothy Wade

United Kingdom

Ann Wales

United Kingdom

Mark Walland

Australia

John Walley

United Kingdom

Lars Wallin

Sweden

Vicky Ward

United Kingdom

Paul Ward

Australia

Margaret Camilla Watson

United Kingdom

Bryan Weiner

United States of America

Elizabeth Whipple

United States of America

Robin Whitebird

United States of America

John Wiggers

Australia

Nancy Wilczynski

Canada

Karen Willis

Australia

Shannon Wiltsey Stirman

United States of America

Kath Wright

United Kingdom

Marieke Zegers

Netherlands

Wen Zeng

Macao

